# Oxidation-Reduction Potential as a Biomarker for Severity and Acute Outcome in Traumatic Brain Injury

**DOI:** 10.1155/2016/6974257

**Published:** 2016-08-25

**Authors:** Kimberly B. Bjugstad, Leonard T. Rael, Stewart Levy, Matthew Carrick, Charles W. Mains, Denetta S. Slone, David Bar-Or

**Affiliations:** ^1^Department of Trauma Research, Swedish Medical Center, Englewood, CO 80113, USA; ^2^InterMountain Neurosurgery, St. Anthony Hospital, Lakewood, CO 80228, USA; ^3^Medical Center of Plano, Plano, TX 75075, USA; ^4^Department of Trauma Research, St. Anthony Hospital, Lakewood, CO 80228, USA; ^5^Department of Biomedical Sciences, Rocky Vista University, Aurora, CO 80134, USA; ^6^Penrose-St. Francis Health Services, Colorado Springs, CO 80907, USA

## Abstract

There are few reliable markers for assessing traumatic brain injury (TBI). Elevated levels of oxidative stress have been observed in TBI patients. We hypothesized that oxidation-reduction potential (ORP) could be a potent biomarker in TBI. Two types of ORP were measured in patient plasma samples: the static state of oxidative stress (sORP) and capacity for induced oxidative stress (*i*cORP). Differences in ORP values as a function of time after injury, severity, and hospital discharge were compared using ANOVAs with significance at *p* ≤ 0.05. Logit regression analyses were used to predict acute outcome comparing ORP, Injury Severity Score (ISS), Abbreviated Injury Scale (AIS), and Glasgow Coma Scale (GCS). Antioxidant capacity (*i*cORP) on day 4 was prognostic for acute outcomes (*p* < 0.05). An odds ratio of 4.08 was associated with poor acute outcome when* i*cORP > 7.25 *μ*C.* I*cORP was a better predictor than ISS, AIS, or GCS scores. sORP increased in those with the highest ISS values (*p* < 0.05). Based on these findings ORP is useful biomarker for severity and acute outcome in TBI patients. Changes in ORP values on day 4 after injury were the most prognostic, suggesting that patients' response to brain injury over time is a factor that determines outcome.

## 1. Introduction

Currently, assessing the extent of injury and prognosticating eventual outcome of patients with traumatic brain injury (TBI) are difficult tasks. The Glasgow Coma Scale (GCS) and the Abbreviated Injury Scale (AIS) correlate with injury severity and have some prognostic power; however they rely on the subjective interpretation of patient injury by health care professionals, cerebral function, and can be influenced by age and other factors [1–6]. For these reasons, the Centers for Disease Control and Prevention has suggested that they are not be used in isolation when assessing the extent of injury or forecasting eventual patient outcome [6, 7].

Biomarkers may provide an unbiased and independent evaluation of TBI severity and patient outcome. Glial fibrillary acidic protein (GFAP), S100*β*, and soluble urokinase plasminogen activator receptor (suPAR) have been used to assess survival in TBI patients [8–10]. Tau and GFAP have also been used to estimate cerebral function using GCS score at time of discharge, and the Glasgow Outcome Scale (GOS) score at 6 or 12 months [4, 8, 11]. Ubiquitin C-terminal hydrolase (UCH-1), matrix metalloproteinase 9 (MMP9), and MMP2 have been associated with TBI severity [12, 13]. Most of these biomarkers can be measured from plasma or serum samples, minimizing the difficulty of obtaining a cerebrospinal fluid (CSF) sample but the process is still not adapted to fast turnaround times in the critical setting of TBI.

A cascade of events that lead to brain tissue ischemia and oxidative stress are evident in TBI. The peripheral and local immune systems respond by releasing proinflammatory cytokines, neutrophils, and reactive oxygen species (ROS); the latter then increases oxidant activity [14–18]. Cytogenic edema and the release of free glutamate potentiate oxidant activity by overwhelming mitochondrial energy through glutamate-induced excitotoxicity and inducing a state of oxidative stress [19, 20]. Thus, a biomarker that associates with oxidative changes in brain injury could assist in the assessment of TBI patients.

Changes in oxidation-reduction potential (ORP), as an indicator of oxidative stress, might be a suitable biomarker for TBI. ORP is the net balance in activity between oxidants and reductants, also known as the redox potential. When oxidant activity exceeds reductant activity, the biological sample is under a state of oxidative stress [21–25]. ORP has been used for over 50 years when determining if the oxidant activity is sufficiently high enough in treated water to kill bacteria and other microbes [26–28]. Early studies in our lab and by others found changes in ORP in blood plasma samples from trauma patients, suggesting the benefit of measuring ORP in the biomedical arena [29–32].

Traditional methods for measuring ORP use large reusable platinum silver-silver chloride electrodes contained within a glass probe which is then submerged into a large volume of sample and analyzed using a galvanometer. The major limitations of this technology have been electrode contamination over time and the large sample volume [33]. Because of these limitations, the application of ORP to biomedical assessment has been limited. A novel technology has removed these limitations by developing a single-use disposable sensor requiring only 30 *μ*L of sample. This new system is sensitive enough to differentiate between oxidative states before and after exercise, to identify septic patients, and to measure changes in antioxidant activity in stored breast milk [34–38]. Using this novel technology, the present study explores the use of ORP as proxy of injury progression, severity, and survival in TBI patients.

## 2. Methods

### 2.1. Participant Information, Ethics, and Consent

Participants were a subset from a retrospective cohort study of multiple trauma/TBI patients previously reported [2]. Trauma patients were admitted to one of two level I trauma centers in the Denver-metro area—Swedish Medical Center (Englewood, CO) or St. Anthony Hospital (Lakewood, CO), between January 1, 2008, and December 31, 2012. The study was approved by the HealthOne HCA and the St. Anthony Hospital institutional review boards. Consent for daily blood draws was given by the patient or their legally authorized representatives. Twenty self-proclaimed healthy individuals were recruited as age-matched controls.

### 2.2. Inclusion Criteria

Patients (*n* = 132) with TBI were identified through the trauma registry (TraumaBase® database, Clinical Data Management) with one or more of these diagnostic injury codes based on the* International Classification of Diseases, Ninth Revision, Clinical Modification *(*ICD-9-CM*): concussion (850.0–850.99); cerebral or cerebellar contusion or laceration (851.0–851.99); subarachnoid hemorrhage (852.0–852.19); subdural hemorrhage (852.2–852.39); extradural hemorrhage (852.4–852.59); other, unspecified intracranial hemorrhage (853.0–853.19); and intracranial injury of unspecified nature (854.0–854.19).

### 2.3. Exclusion Criteria

To identify those in which TBI was the primary injury, patients were excluded if the head AIS was less than two and if another AIS region was scored higher than the head AIS. Patients with less than five plasma samples obtained during hospitalization were also excluded. After exclusions, the final sample size was 104 (Table 1).

### 2.4. Plasma ORP Measurements

Whole blood was collected by venipuncture using heparinized Vacutainers during routine morning blood sampling. Samples were processed to plasma by centrifugation for 10 minutes at 1,000 ×g. Plasma was aliquoted and stored frozen at −80°C until processing. Plasma ORP was measured using the RedoxSYS® system as a measure of the electron transfer from reductants (antioxidants) to oxidants under a constant negligible current (static ORP, sORP) and then by increasing the oxidative current (capacity ORP, cORP). For those interested in additional details regarding the RedoxSYS system, please see Rael et al. [39]. The sORP provides a measure of the current balance between all known and unknown oxidants and reductants/antioxidants. As such, higher sORP (in millivolts, mV) suggests a higher level of oxidative stress. The cORP measures the biological sample's ability to withstand an oxidative insult by applying an increasing oxidizing current. This current ultimately exhausts all antioxidants present in the sample. cORP is expressed in microcoulombs (*μ*C). Typically the higher the cORP, the more the capacity a sample has to mitigate an oxidative insult but, because raw cORP data were not normally distributed, the inverse of each cORP value was generated to normalize the data; thus a* higher i*cORP suggests a* lower* capacity to handle induced oxidative stress. While sORP and cORP are related, one looks at the current state of oxidative stress (sORP), while the other assesses the potential for oxidative stress (cORP).

### 2.5. Study Outcomes

The primary outcome was to determine if ORP could distinguish between discharge status and acute patient outcomes. Acute outcome was dichotomized based on hospital discharge status into good acute outcome (survival: discharged to home, skilled, or acute care facilities) and poor acute outcome (nonsurvival: patients who died or were transferred to hospice) (Table 2). Secondary outcomes included changes in ORP values over time after injury, TBI diagnosis, and degree of severity (Injury Severity Score (ISS) and GCS).

### 2.6. Statistical Analysis

Tables 1 and 2 provide descriptive statistics for the variables and outcomes of interest in the present study. All data were analyzed and graphed using Statistica (Dell, Inc.) or MedCalc. Differences in sORP and* i*cORP between the TBI cohort and aged matched controls were analyzed by Student's* t*-test. Significance level of *p* < 0.05 was used in all analyses. Unless indicated otherwise, all graphs are presented as mean ± SEM.

Differences between discharge statuses were analyzed using ANOVA with Fisher LSD* post hoc* analyses when appropriate. Receiver operating characteristic (ROC) analysis was used to examine acute outcomes in terms of hospital survival based on ORP. The combined prognostic power of ORP, ISS, and AIS-head score was evaluated using forward logistic regression.

To gauge injury progression based on ORP values, data was analyzed for the first 14 days after injury. Data was then grouped into blocks of time after injury to analyze ORP changes long-term: day of injury (day 0, < 24 hours estimated postinjury time), first 48 hours, days 3–7 (week 1), days 8–14 (week 2), days 15–21 (week 3), and ≥21 days (week 4 and beyond). Both analyses were done using one-way ANOVAs.

Group comparisons were used to determine changes in ORP as a function of the type of TBI and the degree of severity defined by ISS and GCS (Table 2). Analyses of ORP measures taken at admission, on day 4 and day 7, were analyzed using separate one-way ANOVAs. Pearson's* r* moment correlation was used between ORP values and ISS, AIS-head, and GCS values.

## 3. Results

### 3.1. Patient Demographics and ED Measures

The antioxidant capacity measures (*i*cORP) taken on day 7 were significantly correlated to the verbal (*r* = 0.31, *p* < 0.05) and motor (*r* = 0.27, *p* < 0.05) portions of the GCS taken at admission, with increasing scores associated with increasing icORP values, thus decreasing antioxidant capacities.* i*cORP values on day 7 and day 4 and sORP values on day 7 were negatively correlated with body temperature on arrival (*r* = −0.43, *r* = −0.37, and *r* = −0.42, *p* < 0.05, resp.), indicating that a higher temperature was associated with a later elevation in antioxidant capacities but also increased oxidative stress. Higher sORP on day 7 was also correlated with higher diastolic blood pressure (*r* = 0.37, *p* < 0.05). ORP measures taken at admission and other day 4 measures were not correlated with ED measures (Table 1). No ORP value was not significantly different based on sex (*p* > 0.05 all days) or whether the patient was ventilated (*p* > 0.05 all days)

### 3.2. Estimating Hospital Discharge Status and Acute Outcome

**Figure 1:**
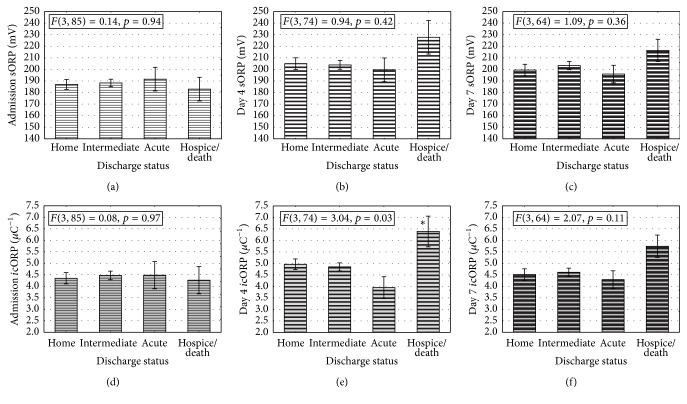
Assessment of survival based on hospital discharge can best be predicted by* i*cORP on day 4. (a) Admission measures of* i*cORP were not significantly different between patients with ultimately different discharge statuses. (b) By day 4 after injury, there was a significant difference between groups, with those that were ultimately discharged to a hospice or who died having much higher* i*cORP values than any other discharge status. Those discharged to acute care facilities were the lowest but this failed to reach significance (*p* = 0.06 and 0.08 between home and intermediate, resp.). (c)* i*cORP values on day 7 were, again, not significantly different between the groups. (d) The ROC curve showing the AUC for day 4* i*cORP values. See Table 3 for ROC results. Data are presented as means ± standard error of the mean (sem); *∗* means significantly greater than all other groups.

The four categories of hospital discharge status were not significantly different in their sORP measures at admission, day 4, or on day 7. Similar* i*cORP levels across discharge statuses were also present at admission; however on day 4 those with a poor acute outcome (those who died or were transferred to hospice) had significantly higher* i*cORP values, and thus lower antioxidant capacity, than those with a better acute outcome (Figure 1). This divergence of groups tended to remain through day 7 although not as strong, as suggested by the trend in significance (*p* = 0.11). Because of the significant effects found on day 4,* i*cORP data were used in ROC and logit regression analyses to determine its strength at predicting a poor or good acute outcome and were compared to the predictive power of ISS, AIS-head, and GCS values.

From the ROC analyses, the area under the curves (AUCs) for day 4* i*cORP values were significant (*p* < 0.05; Table 3). An* i*cORP cut-off value of 7.25 *μ*C generated a specificity of 100%, indicating that all surviving patients had a day 4* i*cORP less than or equal to 7.25 *μ*C. The negative predictive power (NPV) was 97.4% suggesting that in patients with a day 4* i*cORP value less than 7.25 *μ*C it would be highly likely that they have a good acute outcome. ISS and AIS-head scores also had significant AUCs but with lower specificity, negative predictive power, and accuracy (Table 3). GCS scores failed to reach significance in this data set. The logit regression results indicate that only day 4* i*cORP values significantly contributed to the overall model (*X* ^2^ = 5.78, *p* < 0.05) and had an odds ratio of 4.08. AIS-head and ISS values failed to enter the model.

### 3.3. Controls versus TBI and ORP Changes after Injury

**Figure 2:**
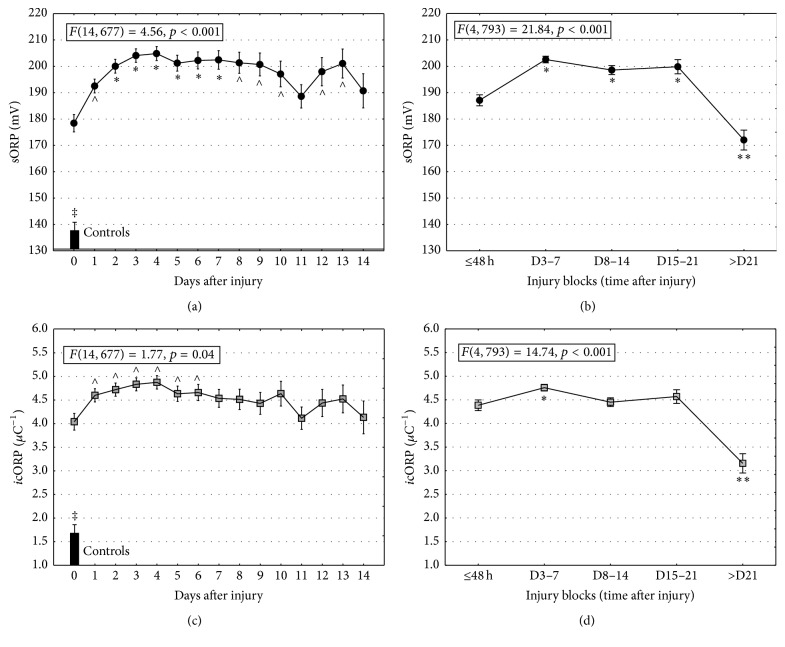
ORP values are higher in TBI than controls and increase in the first few days after injury. (a) Daily changes in sORP indicated significant increases in the first two days after injury and remain reliably high through at least 10 days. Bars represent mean sORP values from healthy controls. (b) Extending the analysis by blocks of time, sORP remains elevated through three weeks (D: days; D15–21). (c) Daily changes in* i*cORP revealed a significant increase (a decrease in antioxidant capacity) in the first day after injury. This steady elevation remains at least through six days after injury. Bars represent mean* i*cORP values from healthy controls. (d) Blocks of data suggested that* i*cORP does not decrease (recovery of antioxidant capacity), until three weeks after injury (D15–21). Data are presented as means ± standard error of the mean (sem); ∧ represents significantly greater than day of injury (day 0); *∗* indicates significantly greater than day of injury and day 1; *∗∗* indicates significantly lower than the first 48 hrs after injury; *p* < 0.05; ‡ means significantly less than TBI.

Plasma samples from healthy controls had significantly lower sORP and* i*cORP values than those measured in TBI patients (sORP: *t*(107) = 9.66, *p* < 0.0001;* i*cORP: *t*(104) = 9.04, *p* < 0.0001; Figure 2).

In the first 48 hours after injury, sORP values increased and remained stable through at least the first 10 days after injury (Figure 2). Grouping the days after injury into blocks of time suggests that sORP values remain stable through at least 21 days and then dropped significantly to levels below those measured in the first 48 hours (Figure 1(b)); however they were still significantly higher than controls levels (*t*(76) = 7.02, *p* < 0.05).

The* i*cORP values revealed a similar pattern. TBI patients experienced an increase in* i*cORP values (lower antioxidant capacity) in the first 24 hours and* i*cORP values remained high through at least 6 days after injury (Figure 2). Antioxidant capacity began to return to more normal values after 21 days after injury; however the* i*cORP values were still higher than those measured in healthy controls (*t*(76) = 4.79, *p* < 0.05).

### 3.4. Injury Severity

On day 4, there were significant correlations between ORP values and the ungrouped ISS, and AIS-head scores (Table 4). Higher ISS and/or AIS-head scores were related to higher sORP and* i*cORP values. GCS scores correlated only with day 7* i*cORP values, with increasing* i*cORP associated with increasing GCS scores. Admission values failed to correlate.

**Figure 3:**
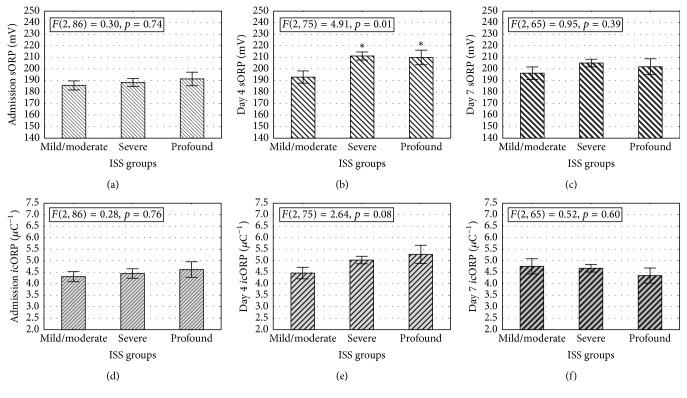
ORP measures increase as a function of ISS groups on the fourth day after injury. (a/d) Admission measures of sORP (a) or* i*cORP (d) did not change as a function of ISS severity. Regardless of severity, groups had similar sORP and* i*cORP values. (b/e) On day 4, sORP measures were significantly higher in the moderate, severe, and profound groups compared to the mild group (b). The severe and profound groups both had increased* i*cORP measures (lowered antioxidant capacity) on day 4 compared to the moderate or mild groups (e). Thus, with increasing level of severity, there were significant increases in sORP and* i*cORP. (c/f) No significant differences were found in ORP measures between moderate, severe, and profound severity groups on day 7 after injury. The mild group did not have enough subjects to be included in this analysis. Data are presented as means ± standard error of the mean (sem); *∗* means significantly greater than the mild severity group; *p* < 0.05.

In confirmation of the correlations, ORP measures on day 4 significantly distinguished between ISS groups (Figure 3). Patients with severe (ISS 16–25) and profound injuries (ISS > 16) had significantly higher sORP measures than the patients classified with mild to moderate injuries (ISS ≤ 15; *p* < 0.05). This effect was not found at admission or on day 7 after injury (Figure 3).

Despite the significant correlation, differences in antioxidant capacity did not reach significance between ISS groups (*p* = 0.08). Patients classified with severe or profound injuries had a trend for higher* i*cORP values than patients with moderate or mild injuries (Figure 3). This effect was not present in* i*cORP values measured at admission or seven days after injury (*p* > 0.05).

### 3.5. Type of TBI Injury

Table 2 presents the TBI diagnoses. Approximately 74.3% of patients had either a subarachnoid, subdural, or epidural hemorrhage. Only two concussion TBI patients had day 7 ORP measures, so this group was not included in day 7 analyses. Analysis of sORP values taken at admission, 4 days after injury, and 7 days after injury did not distinguish between injury types (admission: *F*(4, 81) = 0.84, *p* = 0.50; 4 days: *F*(4, 72) = 0.38, *p* = 0.82; 7 days: *F*(3, 60) = 1.14, *p* = 0.34). Measures of* i*cORP also did not distinguish between groups (admission: *F*(4, 81) = 1.79, *p* = 0.14; day 4: *F*(4, 72) = 0.94, *p* = 0.45; day 7: *F*(3, 60) = 0.21, *p* = 0.89).

## 4. Discussion

The data presented suggests that ORP may be a useful biomarker in TBI. Measures of ORP in TBI patients indicate that after injury they are in a state of oxidative stress, measured by elevated sORP, which is higher in patients with more severe injury. However, and maybe more importantly, is the fact that those patients with an inability to manage the additional oxidative stress, measured by elevated* i*cORP, were more susceptible to a poor acute outcome. Differences in* i*cORP identified, with a high rate of accuracy, the acute outcome of the TBI patients.

Both sORP and* i*cORP increased in the 24–48 hours after injury and remained fairly stable over time regardless of type of TBI, severity, or outcome, except on day 4 where a significant divergence was measured for acute outcome and severity. Patients with a good acute outcome all had a day 4* i*cORP value less than or equal to 7.25 *μ*C. This cut-off value was 96% accurate in identifying patients by acute outcome. Since the time between hospital admission and death/hospice ranged from 6 to 19 days, measuring* i*cORP on day 4 still would provide valuable information about the probability of a good acute outcome several days to weeks in advance. Predicting outcome by GCS, ISS, or AIS-head score was less successful and pairing* i*cORP values with severity scores did not improve their prognostic power. Relative to other published studies,* i*cORP compares favorably with serum-based Tau, suPAR, and Tau; however measuring ORP has the advantage of providing results more quickly [4, 11, 13, 40–45].

TBI severity, measured by ISS and AIS-head score, was correlated with both measures of ORP on day 4. Day 4 appears to be a crucial time point in the resolution of TBI. A limitation of the study is that the patient sample was drawn from those in which TBI was the primary injury but not necessarily their only injury, that is, isolated TBI. Both the mixed state of the injuries and TBI would contribute to altering the redox balance and elevate ORP values. It is possible then that when the lesser injuries begin to resolve, the contribution to redox imbalance made by TBI alone might be detected, allowing us to distinguish between TBI severities and acute outcomes based on ORP levels on day 4.

We further speculate that because brain injury is a multiphasic response, the ORP measured on day 4 captures these later phases and detects potential differences in the ongoing healing process thus allowing us to identify those with a poor acute outcome. The brain's response to TBI unfolds over the course or hours and days; thus the change in day 4* i*cORP values may reflect the point at which current antioxidant reserves begin to falter especially in those patients that have a poor acute outcome. Based on controlled animal experiments in TBI, we can establish a timeline of events which may contribute to the depletion of those antioxidant reserves. There is an immediate disruption of the BBB after injury along with the accumulation of neutrophils and the expression of MMP9, myeloperoxidase, and the anti-inflammatory cytokine, IL-10 [16, 17, 46–50]. Many of these elevations will decline over the course of several days after injury. Others, like IL6, IL8, MMP2, TNF-p55, and TNF-p75 and the invasion of reactive astrocytes emerge in subsequent days sustaining the state of oxidative stress [15, 16, 48, 50]. In addition, the BBB disruption and invading neutrophils have a biphasic response, which could reinitiate the inflammatory reaction and the ensuing oxidative stress [16, 17, 46, 47]. The time course for changes in sORP and* i*cORP parallels, especially in the initial days after injury, the emergence of free-radicals, elevated cytokine expression, and neutrophil recruitment.

## 5. Conclusion

The current study supports the notion that ORP can be a valuable tool in assessing injury severity and acute outcome in TBI. A difference in antioxidant capacity, measured by* i*cORP, appears to differentiate between those that will have a good acute outcome from those that will not. sORP, an all-inclusive measure of oxidative stress, was a valuable indicator of injury severity. Interestingly, the projective powers of both sORP and* i*cORP were found in samples taken four days after injury, emphasizing the importance of the bodily response to injury with time. The types of TBI present in the patient sample had similar levels of ORP.

## Figures and Tables

**Table 1:** Participant demographics.

Demographic	Mean (95% CI)	Median (IQR)	*N*
Age (years)	54.53 (50.0–59.0)	55.5 (34–76)	104
AIS-head score	3.85 (3.7–4.0)	4 (3–5)	104
ISS	18.77 (17.4–20.2)	17 (14–25)	104
GCS	11.27 (10.3–12.3)	14 (7–15)	91
ICU LOS	6.18 (5.1–7.3)	4.5 (2–9)	104
Hospital LOS	12.51 (10.6–14.4)	10 (6.5–15)	104
ED, pulse	86.81 (83.0–90.7)	86 (73–96)	102
ED, temperature (°F)	97.60 (97.1–98.0)	97.8 (96.8–98.4)	45
ED, respiratory rate	16.77 (15.5–18.0)	16.5 (14–20)	102
ED, systolic BP	139.38 (134.5–144.2)	134 (124–154)	102
ED, diastolic BP	79.79 (76.0–83.60)	82.5 (71–89)	52
ED, probability of survival	0.86 (0.82–0.91)	0.96 (0.83–0.98)	91
sORP at admission	187.78 (183.0–192.5)	185.9 (172.2–205.2)	89
*i*cORP at admission	4.42 (4.2–4.7)	4.46 (3.5–5.3)	89

Gender (ratio females : males)	37 : 67		104
Number of complications (%)			104
* None*	*64.1%*		*67*
* One*	*21.94%*		*23*
* Two–five*	*13.9%*		*14*

ED: emergency department; LOS: length of stay.

**Table 2:** Group categories used in statistical analyses.

	*N*
*Type of TBI injury*	
Concussion (*any participant with a concussion*)	4
SAH w/SDH/EDH (*subarachnoid hemorrhage with any other hemorrhage*)	37
SAH alone	23
SDH/EDH (*subdural hemorrhage and/or epidural hematoma*)	15
Other (*skull fracture, other non-ICH injury, anything else*)	22

*Total*	*101*

*ISS severity category*	
Mild/moderate (*ISS score < 15*)	33
Severe (*ISS score 16–25*)	51
Profound (*ISS score > 25*)	20

*Total*	*104*

*GCS category*	
Minor (≥13)	57
Moderate (12–9)	8
Severe (≤8)	26

*Total*	*91*

*Hospital discharge status*	
Good acute outcome	
Home (*home or home health*)	31
Intermediate (*skilled nursing or rehabilitation facilities*)	57
Acute (*long-term acute care*)	10
Poor acute outcome	
Death/hospice (*min–max hospital LOS: 6–19 days*)	6

*Total*	*104*

**Table 3:** Comparative values of the ROC analyses for* i*cORP on day 4, ISS, AIS-head, and GCS at predicting acute outcome.

	icORP day 4	ISS	AIS-head	GCS
*Cut-off for survival status*	≥*7.25 μC*	*>27*	*>4*	≤*8*

ROC-AUC	0.87^*∗*^ (*0.78–0.94*)	0.78^*∗*^ (*0.68–0.85*)	0.73^*∗*^ (*0.63–0.81*)	0.54 *(0.43–0.65)*
Sensitivity (% of poor acute outcomes)	33.33 (*0.8–90.6*)	33.33 (*4.3–77.7*)	50.0 (*11.8–88.2*)	40.0 *(5.3–85.3)*
Specificity (% of good acute outcomes)	100 (*95.2–100*)	89.90 (*82.0–95.0*)	75.51 (*65.8–83.6*)	72.1 *(61.4–81.2)*
Positive predictive value	100 (*2.5–100*)	16.7 (*2.1–48.4*)	11.1 (*2.4–29.2*)	7.7 * (0.9–25.1)*
Negative predictive value	97.4 (*90.9–99.7*)	95.7 (*89.2–98.8*)	96.1 (*89.0–99.2*)	95.4 *(87.1–99.0)*
Accuracy	96%	87%	74%	70%
*N*	78	104	104	91

95% confidence intervals. ^*∗*^Significant AUC values, *p* < 0.05.

**Table 4:** Correlations between sORP and* i*cORP and traditional measures of severity.

	sORPs			*i*cORP		
	Admission	Day 4	Day 7	Admission	Day 4	Day 7
ISS	0.11	0.36^*∗*^	0.15	0.05	0.28^*∗*^	−0.08
AIS-head	0.10	0.36^*∗*^	0.16	0.08	0.28^*∗*^	0.00
GCS	−0.01	−0.18	0.13	−0.01	−0.04	0.29^*∗*^

^*∗*^Significant correlation; *p* < 0.05.
